# Advances in innovative exosome-technology for real time monitoring of viable drugs in clinical translation, prognosis and treatment response

**DOI:** 10.18632/oncotarget.27927

**Published:** 2021-05-25

**Authors:** Mujib Ullah, Nicole Pek Min Qian, Gustavo Yannarelli

**Keywords:** exosomes, artificial intelligence, drug delivery, extracellular vesicles, cancer

## Innovative technologies and exosomes

In the field of medicine, technological discovery is a vital way to bridge knowledge gaps and equip us with the know-how to address biological challenges. Innovative technologies allow us to work faster and better understand complexities, especially pertaining to human health and disease [1–3]. Computer simulation and artificial intelligence play a significant role in the timely diagnosis and effective treatment of complex ailments such as cancer [2–5]. The inquisition towards developing and acquiring new technologies is quintessential in the journey towards improving the quality of patient care.

The advent of advanced exosome purification methods has made it possible to tap on the unexplored potential of these tiny particles in clinically-precise diagnosis and treatment of a myriad of diseases [6–8]. More efforts are currently being funneled into research and development endeavors in order to increase the quality and reach of exosomes-based diagnostic and therapeutic applications in the near future.

Exosomes are small nano-particles made by cells within our body [8–10]. They contain crucial information by way of proteins, metabolites, and nucleic acids, facilitating cell-cell communication cells [8–10]. Structurally, exosomes are surrounded by lipid bilayers, which provides a robust layer of protection to the biological contents stored within [10]. The abundance of adhesive and surface proteins found on the surface of exosomes readily interact with the cellular membrane of target cells, allowing exosomes to essentially be vehicles to deliver cargo such as drugs [10–12]. The innovative use of exosomes as drug delivery systems for small molecules, cytokines, and other biological components makes them an ideal choice for clinical use [13].

In addition, the recent COVID19 pandemic has opened up new opportunities for exosome technology to benefit humankind. In the realm of vaccine development, researchers are creating vaccines that consist of exosomes engineered to display SARS-CoV-2 proteins on their surface [14–16]. Also, since exosomes are known to be resistant to immune attack, lysis, and degradation [13, 16–18], they can be exploited for use as an early-stage diagnostic marker. Many conventional biomarkers that are often proteinaceous-in-nature are unprotected from biofluids which are home to degradative enzymes such as proteases [17, 19]. Exosomes, which are encapsulated within lipid bilayers, will be able to withstand said harsh conditions and are stable for a prolonged duration of time. Therefore, exosome-based biomarkers are attractive options for efficient diagnosis of diseases such as cancer.

Other benefits that could give exosomes the edge over existing platforms are that they are less immunogenic, less cytotoxic, and due to sheer size, can easily cross biological barriers such as the blood–brain barrier (BBB) [19–21].

**Figure 1:**
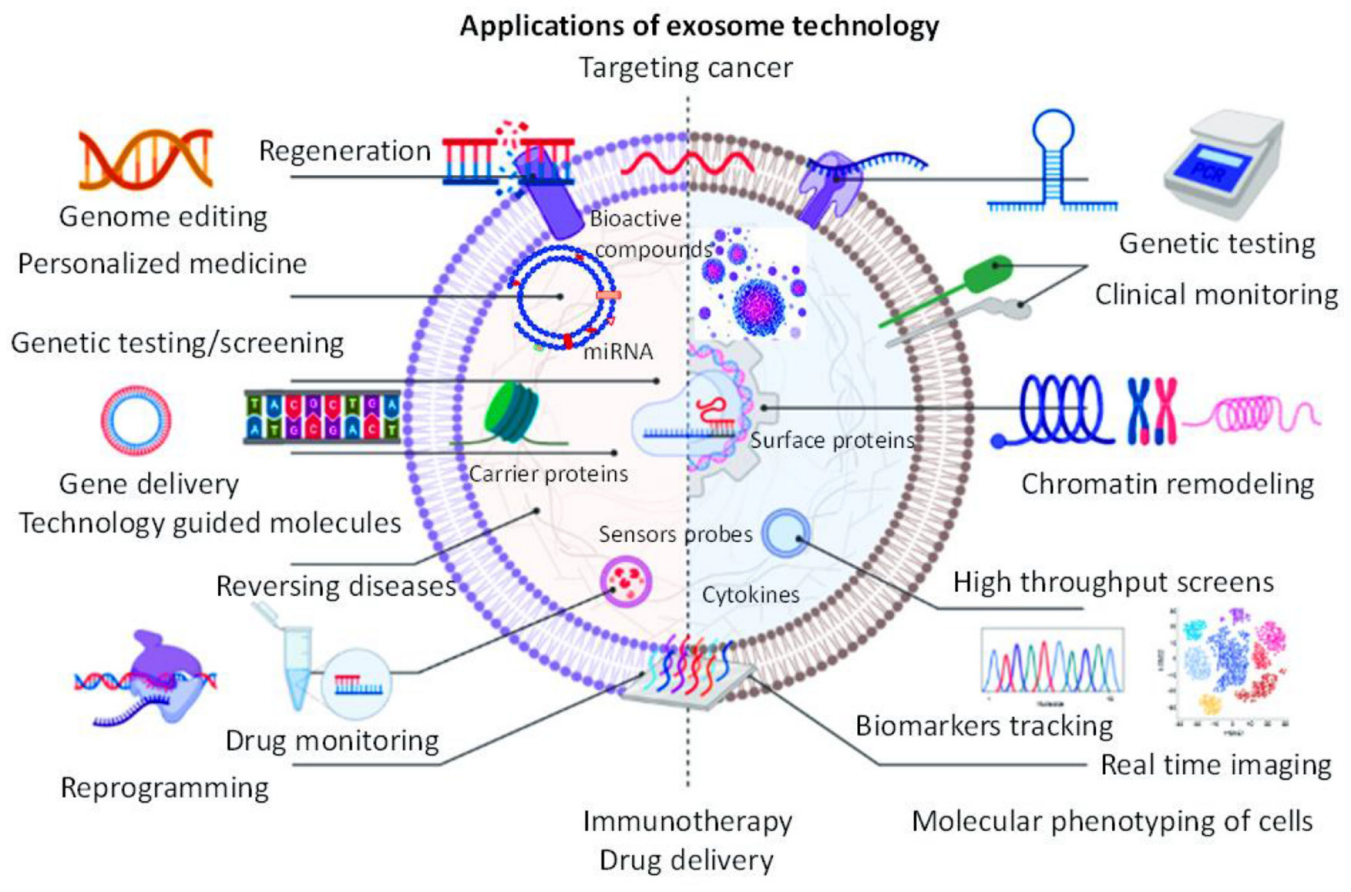
Schematic illustration showing the application of exosome technology in major clinical uses and in different scientific investigation.

Innovations pertaining to the engineering of exosomes have progressively circumvented the limitations of naive exosomes (naturally-occurring exosomes produced by cells), which are known to have lower targeting capabilities, are more challenging to isolate, low drug loading capacities, bioavailability issues, low half-life in circulation, and low concentration of functional surface receptors [22–24]. Engineered exosomes are manipulated in a way that they can be loaded with maximum amounts of components such as drugs or miRNAs, and have higher specificity and delivery efficacies [13, 24, 25]. Additionally, engineered exosomes have other advantages over native exosomes, such as lower production cost, higher biocompatibility, and low undesirable effects such as cytotoxic effects [25, 26]. No doubt, the clinical application of exosomes can be enhanced by intelligent engineering methods for improving the delivery of drugs, genes, heat shock proteins, and functional bioactive molecules to target tissues [26–28]. They also have the capabilities to penetrate deeper within tissues with their surface ligands and receptors (Figure 1) [27, 28].

## Conclusions and Future Directions

New and emerging technologies play a significant role in permitting exosome detection and tracking, in vivo monitoring and analysis [29–31]. Magnetically-labeled particles and optical sensors offer great promise for in vivo monitoring, and detection of exosomes [31, 32]. Innovative technologies are also beneficial to improve exosome target specificity, which translates to higher sensitivity of patients to drug response, reduction in treatment cost, and greater disease outcome [31–33]. Artificial, digital, and optical technologies, when used in combination, have the potential to detect exosomes at the single-particle level or at very minimal concentrations, which have a high diagnostic value [33–36]. Collectively, exosome technology will revolutionize the field of diagnostics and treatment of human diseases (Figure 1).

Nonetheless, further optimization of current methods and fine-tuning of technologies are necessary to push the field towards real clinical value [37, 38]. Exosomes technology has already been increasingly used in disease diagnosis and treatment; this encourages researchers to channel more efforts into overcoming limitations and challenges in the field [38, 39] such as issues related to the purification of exosomes from their parent source, eliminating contaminants, reducing the damage of exosomes from the harsh ultracentrifugation method (which spins the exosomes at ultra-speed and damages their membrane and enclosed therapeutic content) [39–42].
